# Investigating the Impact of Alexithymia and Dissociation on the Severity of Symptoms in Severe Personality Disorders

**DOI:** 10.1192/j.eurpsy.2025.1932

**Published:** 2025-08-26

**Authors:** L. Ghidetti, S. Costantini, D. Salmaso, M. Benassi, R. P. Sant’Angelo, M. Pacetti

**Affiliations:** 1 Psychology, University of Bologna; 2 Mental Health, Ausl Romagna, Cesena; 3 Mental Health Department, Ausl Romagna, Forlì; 4 Mental Health Department, Ausl Romagna, Cesena, Italy

## Abstract

**Introduction:**

Severe personality disorders are characterized by deeply ingrained patterns of thought, behavior, and emotional functioning, along with impaired mental functioning in the areas of self-stability and identity formation. The challenges in interpersonal relationships and overall functioning result in a significant reduction in the capacity to adapt to social roles.

**Objectives:**

In this context, the present study aims to explore the severity of symptoms (SPrDP) in correlation with alexithymia (TAS-20) and dissociation (DES II) in individuals under the care of the Department of Mental Health of Forlì-Cesena.

**Methods:**

The sample was selected using the SCID PD (Structured Clinical Interview for DSM-5), followed by the administration of the following scales: SPrDP (Parma’s Scale), TAS-20 (Toronto Alexithymia Scale), and DES II (Dissociative Experiences Scale). Multivariate analyses were applied, including non-parametric correlations (Spearman’s Rho) between variables using SPSS software.

**Results:**

The sample consists of 55 individuals (F=38; M=17), of whom 42 have borderline personality disorder (BPD), 3 have narcissistic personality disorder (NPD), 6 have histrionic personality disorder (HPD), 3 have obsessive-compulsive personality disorder (OCPD), and 1 has dependent personality disorder (DPD). The analysis did not reveal a significant correlation between SPrDP and the TAS-20 and DES II scales, although a trend was observed that could correspond to more compromised personality functioning in the presence of high scores on these scales. Furthermore, a statistically significant correlation was found between TAS-20 and DES II. Higher marginal means for TAS-20 were observed in individuals with OCPD. For DES II, the highest estimated marginal means were found in individuals with OCPD and BPD.

**Conclusions:**

The symptom severity measured by SPrDP was not statistically significant in relation to TAS-20 and DES II. However, the presence of a trend between the scales suggests the possibility of further investigation with a larger sample. Additionally, the statistically significant correlation between alexithymia and dissociation may indicate that difficulties in identifying and managing intense emotions lead to the use of dissociative defense mechanisms. The higher average alexithymia scores in individuals with OCPD suggest a possible emotional rigidity with a tendency to control or suppress emotions. The frequency of dissociative symptoms observed in individuals with BPD and OCPD may indicate the centrality of alexithymia and dissociation in the impairment of emotional and relational functioning in severe personality disorders. Further studies are necessary.

**Disclosure of Interest:**

None Declared

